# Predictors of recurrence after vesicovaginal fistula repair: a systematic review of surgical and patient-related factors

**DOI:** 10.3389/fruro.2026.1878604

**Published:** 2026-06-23

**Authors:** Mohammad Shazib Faridi, Lubna Inam, Vyomesh Rastogi

**Affiliations:** 1 Department of Urology, Dr. Baba Saheb Ambedkar Medical College and Hospital, New Delhi, India; 2 Department of Obstetrics & Gynaecology, Hamdard Institute of Medical Sciences and Research, New Delhi, India

**Keywords:** failure, prognostic factors, recurrence, surgical procedure, systematic review, vesicovaginal fistula

## Abstract

**Background:**

Repair of vesicovaginal fistula (VVF) is highly effective, with primary success rates of 80-95%; however, recurrence is reported in 10-30% of cases. Factors influencing recurrence include fistula size and tissue characteristics.

**Methods:**

This systematic review identifies predictive factors for VVF recurrence by reviewing 21 studies published between 1994 and 2025 that met PRISMA criteria, and by analyzing the surgical outcomes from PubMed/MEDLINE data for various etiologies of VVF. Eligible studies reporting predictors of VVF repair were assessed for bias using the modified Newcastle-Ottawa Scale.

**Results:**

The following factors were consistently identified as predictors of VVF recurrence: fistula size greater than 2-3cm (odds ratio [OR]: 1.0-6.0), severe peri-fistula fibrosis (OR: 2.7 to 12.0), involvement of the urethra and/or bladder neck (OR: 0.4 to 9.0) and multiple fistulas (OR: 4.0 to 8.0). The following protective factors were identified: early intervention, surgery performed in a specialist center, and the use of interposition flaps. The Goh and Panzi classifications assist in the predictive risk stratification of patients. Most studies had a low to moderate risk of bias. Due to the heterogeneous nature of the studies, there was considerable variation in study design, etiology, and surgical technique; therefore, narrative synthesis rather than meta-analysis was performed. In most cases, secondary VVF repair has been documented to improve surgical outcomes; however, results for previously failed repairs remain contradictory.

**Conclusion:**

Using validated predictive factors for preoperative risk stratification may improve global VVF surgical outcomes. Closing the gap in surgical outcomes between regions and optimizing surgical techniques will require further prospective research and the development of predictive models.

## 1 Introduction

A vesicovaginal fistula (VVF) is an abnormal epithelialized communication between the bladder and the vagina that leads to continuous involuntary urinary leakage through the vagina (1). This distressing condition profoundly affects physical, psychological, and social well-being, resulting in chronic urinary incontinence, recurrent infections, shame and social ostracization, significantly impacting the quality of life (2). Globally, more than three million females worldwide live with untreated VVF with 30,000-130,000 new obstetric fistulas occur annually in Africa alone (3). The prevalence of VVF following hysterectomy is 0.8 to 1.0 per 1000 women (4, 5).

The etiology of VVF varies significantly across regions. In LMICs, prolonged obstetric labor predominates (6) whereas in high-income nations and urban tertiary centers, iatrogenic causes such as pelvic surgery, particularly hysterectomy is the leading contributor (3). Radiation therapy and pelvic malignancies also contribute to the rising incidence of VVF (7). These etiologic and tissue-quality differences influence fistula anatomy and the likelihood of successful closure (7).

While modern surgical techniques achieve primary closure rates of 80–95%, recurrence or failure after repair remains a significant challenge, with reported rates of 10- 30% (8–10). Although various studies have reported multiple predictors for recurrence of VVF repair such as large fistula diameter, multiple tracts, urethral involvement, previous repair, and active infection (11, 12), but heterogeneity in how “recurrence” or “failure” is defined further limits the comparability.

The present systematic review aims to identify and synthesize surgical and patient-related determinants of recurrence after vesicovaginal fistula repair.

## 2 Methods

### 2.1 Study design

This systematic review was conducted to identify the predictors of recurrence following surgical repair of vesicovaginal fistula (VVF). The reporting and conduct followed the Preferred Reporting Items for Systematic Reviews and Meta-Analyses (PRISMA) guidelines (13). The protocol for this review is registered with The Open Science Framework (OSF) at https://doi.org/10.17605/OSF.IO/RPZG8.

### 2.2 Search strategy

A comprehensive search of PubMed/MEDLINE was conducted to identify English-language studies of surgical VVF repair reporting recurrence or predictors of recurrence. The search combined Medical Subject Headings (MeSH) and free-text terms using Boolean operators. The final search string included terms were: (“vesicovaginal fistula” [MeSH Terms] OR “vesicovaginal fistula” [Title/Abstract] OR VVF [Title/Abstract]) AND (recurrence [MeSH Terms] OR recurrence [Title/Abstract] OR recurrent [Title/Abstract] OR “surgical failure” [Title/Abstract] OR “treatment outcome” [MeSH Terms]) AND (surgical[Title/Abstract] OR repair [Title/Abstract] OR martius[Title/Abstract] OR flap[Title/Abstract] OR transvaginal [Title/Abstract] OR transabdominal[Title/Abstract] OR laparoscopic[Title/Abstract]) OR robotic[Title/Abstract]) AND (predict*[Title/Abstract] OR determinant*[Title/Abstract] OR “risk factor”[MeSH Terms] OR “risk factor”[Title/Abstract]) AND english [Language] AND (“1994/01/01”[Date - Publication]: “2025/12/31” [Date - Publication]) AND humans[MeSH Terms].

The search was limited to 1994–2025 and human studies in English-language. Additionally, the reference lists of included articles were manually screened to identify additional eligible reports.

### 2.3 Inclusion and exclusion criteria

We included peer-reviewed primary studies (prospective or retrospective cohort studies, cross-sectional analyses and case series) that documented surgical repair of VVF in humans, quantified surgical outcomes (closure, recurrence or failure), and evaluated the predictors, determinants, or risk factors for recurrence. The eligible studies were published in English between 1995 and 2025.

We excluded studies that focused on non-surgical management of VVF, non-documentation of recurrence or surgical outcome data or were reviews, case reports, editorials, letters, or conference abstracts without primary patient data. Studies with insufficient methodological detail were also excluded.

### 2.4 Study selection and data extraction

**Figure 1:**
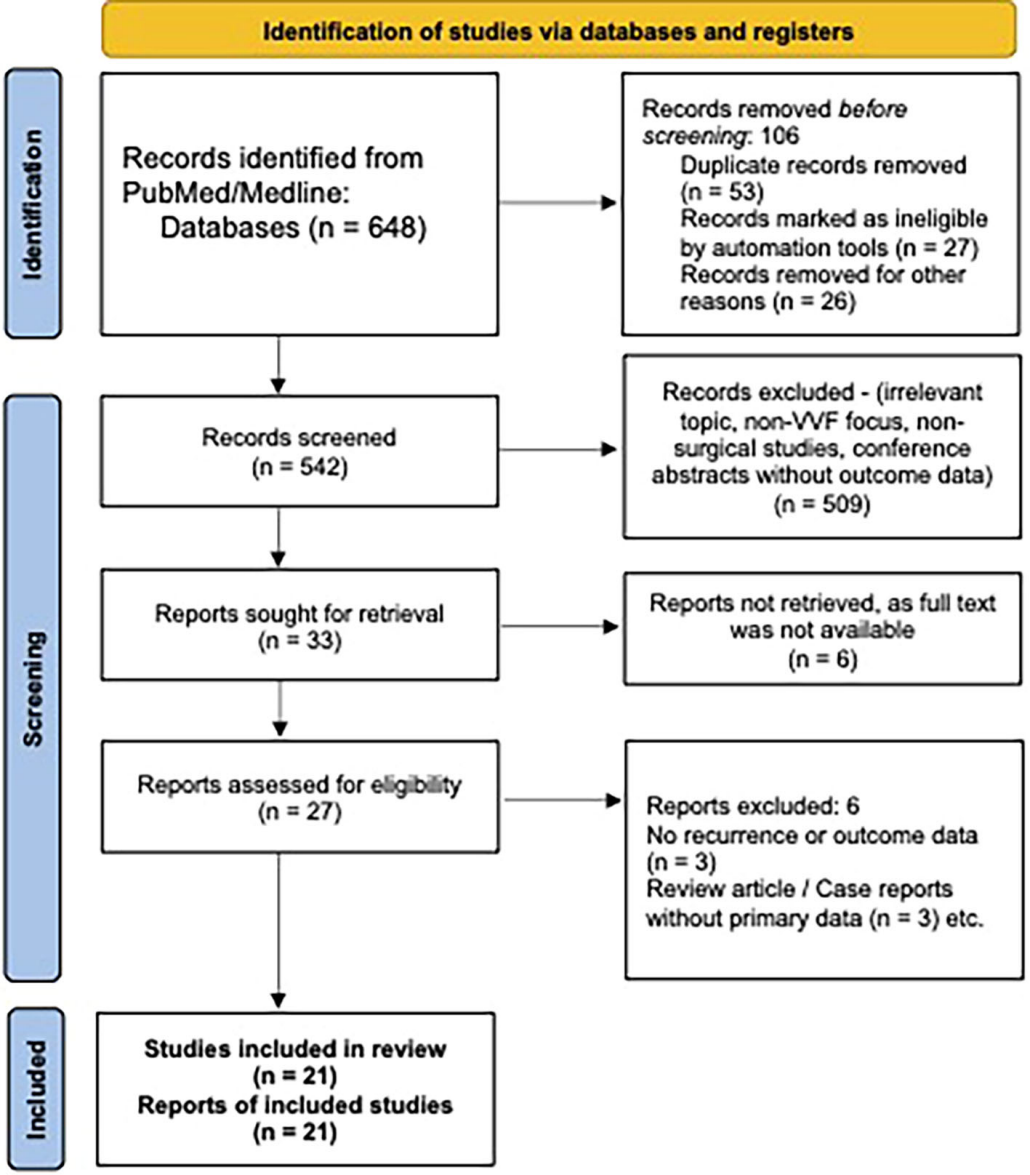
PRISMA 2020 flow diagram for systematic reviews based on searches of databases, registers, and other sources.

A total of 648 studies were identified through database and manual searches. After the removal of 106 records (duplicates, ineligible by automation tools), 542 studies were screened. Following the title and abstract screening, 509 studies were excluded for being non-English language, irrelevance, non-VVF focused, non-surgical management or lack of outcome data. Furthermore, six studies were excluded due to non-availability of full text. Hence, 27 studies were assessed for eligibility; 6 were eliminated for lack of recurrence data or were review articles or case reports without primary data. Finally, 21 studies met the inclusion criteria and were incorporated into the qualitative synthesis (**Figure 1**).

Data were extracted using a standardized data collection form. The extracted variables included year of publication, study design and setting, sample size and patient characteristics, fistula etiology (obstetric, iatrogenic, radiation-related), surgical approach (transvaginal, transabdominal, laparoscopic), use of interposition techniques, measures of surgical/functional success (closure rates), recurrence or failure rates and predictors for determinant of recurrence.

### 2.5 Quality appraisal

The two reviewers independently assessed the methodological quality of included studies, assessed using the Newcastle–Ottawa Scale (NOS) adapted for surgical outcome studies (14). The NOS was initially developed for cohort and case-control designs. Consequently, for case series, the relevant domains were applied pragmatically and the overall risk of bias was assessed with consideration to study design-specific limitations. Discrepancies were resolved by discussion. Studies were categorized as low, moderate, or high risk of bias based on their methodological performance (selection, comparability and outcome domains).

## 3 Results

### 3.1 Study selection

This systematic review analyzes 21 studies published from 1994 and 2025 that evaluated predictors of recurrence following vesicovaginal fistula repair. As the included studies differed substantial in design, patient populations, fistula etiologies, surgical techniques, and outcome reporting, a quantitative meta-analysis was not feasible. Therefore, a qualitative narrative synthesis was performed.

### 3.2 Study characteristics

Despite variation in geographic locations, etiology, and surgical contexts, the overall primary repair success rates were consistently high, exceeding 80–95% (8–10). However, a subset of patients remained at increased risk of repair failure. The findings from retrospective cohorts, prospective studies, and large programmatic series strengthens the external validity of the identified risk factors despite heterogeneity in design and follow-up.

Etiologic patterns differed by regiona: obstetric fistulas predominated in low-resource areas, while iatrogenic (post-hysterectomy) and radiation-induced cases prevail in high-income settings (3). The most consistent reported predictors of recurrence were fistula size, prior repair, fibrosis, urethral involvement, and a history of radiation. Risk-stratified approaches, standardized classification, and predictive models can optimize outcomes. Despite methodological heterogeneity, specific predictors of recurrence were consistently identified.

### 3.3 Predictors of recurrence and poor outcomes

Across the studies, certain predictors of recurrence or poor surgical outcome were consistently identified. The most common risk factors were large fistula size, multiple prior repair attempts, greater degree of fibrosis, urethral or bladder neck involvement, longer duration of fistula, higher complexity of the fistula on classification systems (for example, Goh or Panzi). Other adverse factors included urinary tract infection, multiple fistulas, and comorbid conditions.

The protective factors identified were early intervention, surgeon expertise, management at specialized fistula clinics and careful patient selection (**Table 1**).

**Table 1:** Predictors and outcomes of vesicovaginal fistula (VVF) repair.

S.No	Author	Year	Study design	Surgical approach	Outcomes (closure / failure)	Predictors of failure	Key remarks	Follow up duration	Recurrence definition	Multivariate analysis (Yes/No)
1	Arrowsmith SD (19)	1994	Case series	Genitourinary reconstruction techniques	Not specified	Fistula size, tissue quality	Early reconstructive principles	Post operative period only	Not defined	No
2	Rangnekar NP et al. (31)	2000	Retrospective	VVF repair with Martius flap	High success (~90%)	Poor vascularity, complex fistula	Highlighted role of Martius interposition	6–66 months	Not defined	No
3	Ayed M et al. (15)	2006	Retrospective	VVF repair	Recurrence predictors analyzed	Large size, prior repair, scarring	Identified prognostic factors	6–59 months	Not defined	Yes
4	Chigbu CO et al. (29)	2006	Retrospective	Vaginal vs abdominal repair	Variable outcomes	Juxtacervical fistula location	Compared repair routes	Not mentioned	Not defined	No
5	Goh JT et al. (27)	2008	Prospective classification study	Obstetric fistula repair	Failure prediction model	Goh classification stage	Introduced predictive classification	Not mentioned	Not defined	Yes
6	Lewis A et al. (23)	2009	Retrospective	Genitourinary fistula repair	Closure ~85–90%	Large fistula, prior surgery	505-case Sierra Leone series	3 months	Not defined	Yes
7	Ockrim JL et al. (7)	2009	Retrospective	VVF & UVF repair (mainly vaginal)	Success ~80–90%	Radiation, prior repair, size	Developed-world tertiary data	3 monthly	Continuous urinary leakage at 6 months follow up	No
8	Nardos R et al. (22)	2009	Retrospective	Vaginal obstetric VVF repair	Failure ~10–20%	Urethral involvement, size	Obstetric fistula population	Not Mentioned	Not defined	Yes
9	Sjøveian S et al. (18)	2011	Retrospective	Vaginal repair	Closure ~85–90%	Previous repair, scarring	Large African cohort	Not Mentioned	Not defined	Yes
10	Kayondo M et al. (17)	2011	Prospective	Vaginal repair	Failure ~10–15%	Duration of fistula, fibrosis	Uganda referral center	Not Mentioned	Not defined	Yes
11	Miklos JR & Moore RD (30)	2015	Case series (15-year experience)	Laparoscopic extravesical VVF repair	High success (~95%)	Complex fistula, prior repair	Demonstrated feasibility of laparoscopic minimally invasive repair	3–64 months	Not defined	No
12	Loposso M et al. (20)	2016	Retrospective	Obstetric fistula repair	Recurrence ~10–15%	Prior surgery, complexity	Predictors analyzed	3 months	Continuous urinary leakage after removal of urinary catheter	Yes
13	Zhou L et al. (8)	2017	Retrospective	VVF repair (139 cases)	Outcomes evaluated	Size, prior repair, etiology	Outcome determinant study	3 months	Not defined	Yes
14	Beardmore-Gray A et al. (11)	2017	Retrospective	VVF repair	Outcome varies by stage	Goh classification stage	Validated classification	Not Mentioned	Not defined	No
15	Mukwege D et al. (28)	2018	Cross-sectional	Surgical repair	Severity-based outcomes	Panzi severity score	Introduced scoring model	Not mentioned	Not defined	Yes
16	Bernard L et al. (26)	2019	Retrospective	Obstetric fistula repair	Failure predictors analyzed	Large size, scarring, repeat repair	Angola cohort	Not mentioned	Not defined	Yes
17	Colenbrander J et al. (25)	2021	Case series	Vaginal repair	Closure ~90%	Complex fistula	Supports vaginal approach	3 months	Not defined	No
18	Zaghbib S et al. (16)	2021	Retrospective	VVF repair	Predictors analyzed	Large fistula, prior repair	Tunisia epidemiology	6 months	Continuous urinary leakage after removal of urinary catheter	No
19	Maljaars LP et al. (21)	2023	Retrospective	Repeat fistula surgery	Higher failure rate	Multiple previous repairs	Focus on repeat surgery	Not Mentioned	Not defined	Yes
20	Chaker K et al. (24)	2025	Prospective	VVF repair	Model-based failure prediction	Clinical risk variables	Prediction tool development	6 months	Not defined	Yes
21	Zeleke LB et al. (12)	2025	Prospective	Obstetric fistula repair	Closure ~85–90%	Size, duration, prior repair	Ethiopia multicenter study	Not Mentioned	Not defined	Yes

TAH, Total Abdominal Hysterectomy; VVF, Vesicovaginal Fistula.

### 3.4 Risk of bias assessment

Using the Newcastle–Ottawa Scale, the methodological quality of the included studies showed a low to moderate risk of bias. Most studies were retrospective cohorts or case series that had adequate case definition and selection but limited adjustment for confounders such as fistula size, previous repair, fibrosis, and etiology. However, more recent prospective cohort and predictive-modeling studies published after 2017 showed a lower risk of bias due to improved study design, better control of confounding variables, and more structured outcome assessment. Higher risks of bias were evident in small case series, studies with indirect outcomes or short study up, and those from tertiary referral centers (potential referral bias). Nevertheless, all studies used a similar primary endpoint- anatomical fistula closure. Selection was scored out of 4 stars, comparability out of 2, and outcome assessment out of 3; studies scoring 7–9 stars were categorized as low risk of bias, 5–6 stars as moderate risk, and <5 stars as high risk” (15) (**Table 2**).

**Table 2:** Risk of bias assessment (modified Newcastle–Ottawa scale).

Study (Author, Year)	Selection (Representativeness, Case definition)	Comparability (Confounder control)	Outcome (Assessment, follow-up)	Overall RoB	Key bias concerns
Arrowsmith SD, 1994 (19)	★★★	★	★★	Moderate	Early case series; limited comparator data; short follow-up
Rangnekar NP et al., 2000 (31)	★★★	★★	★★	Low	Retrospective design; single-center surgical experience
Ayed M et al., 2006 (15)	★★★	★★	★★	Low	Retrospective design; variable follow-up
Chigbu CO et al., 2006 (29)	★★★	★	★★	Moderate	Route comparison but limited adjustment for confounders
Goh JT et al., 2008 (27)	★★★	★★	★★	Low	Classification model; limited external validation initially
Lewis A et al., 2009 (23)	★★★	★★	★★★	Low	Large cohort but retrospective program data
Ockrim JL et al., 2009 (7)	★★★	★★	★★	Low	Retrospective; tertiary referral bias
Nardos R et al., 2009 (22)	★★★	★★	★★★	Low	Obstetric-only cohort; fibrosis grading variability
Sjøveian S et al., 2011 (18)	★★★	★★	★★★	Low	Large cohort but retrospective design
Kayondo M et al., 2011 (17)	★★★	★★	★	Moderate	Resource-limited setting; follow-up variability
Miklos JR & Moore RD, 2015 (30)	★★	★★	★★★	Low	Case series of laparoscopic repairs; selection bias
Loposso M et al., 2016 (20)	★★★	★★	★★★	Low	Recurrence-focused retrospective study
Zhou L et al., 2017 (8)	★★★	★	★★	Moderate	Mixed surgical techniques; heterogeneity
Beardmore-Gray A et al., 2017 (11)	★★★★	★	★★★	Low	Classification validation; limited confounder adjustment
Mukwege D et al., 2018 (28)	★★★★	★★	★	Low	Cross-sectional severity scoring; outcome linkage limited
Bernard L et al., 2019 (26)	★★★	★★	★★	Low	Obstetric cohort; repeat surgery bias
Colenbrander J et al., 2021 (25)	★★	★	★★★	Moderate	Case series; small sample size
Zaghbib S et al., 2021 (16)	★★★	★★	★★★	Low	Retrospective regional cohort
Maljaars LP et al., 2023 (21)	★★★	★	★★	Moderate	Repeat surgery cohort; referral bias
Chaker K et al., 2025 (24)	★★★★	★★	★	Low	Predictive modeling study; external validation pending
Zeleke LB et al., 2025 (12)	★★★	★	★★★	Low	Multicenter variability

“Studies scoring 7–9 stars were categorized as low risk of bias, 5–6 stars as moderate risk, and <5 stars as high risk.".

### 3.5 Outcome definition

The outcome definitions varied across studies. Some described the “successful” as complete anatomical closure of VVF without any incontinence (3, 16–18), while others defined success as anatomical closure with or without objective/subjective urinary incontinence (19, 20), or simply as absence of on testing (21, 22). The definitions of ‘failure’ also varied, including failed anatomical closure (23) or persisting urine leakage on dye test and cystoscopy (8). The definition of ‘recurrence’ differed as well, ranging from continuous urinary leakage at 6 months follow up (7) to immediate post-operative leakage reported by some authors (21, 24). Timing of outcome assessment ranged from discharge (2–3 weeks post repair) (18, 25) to 3 months (8) or 6 months (7). The variability in outcome definitions and timing explains the differences in reported recurrence rates and the effect of predictors across studies.

## 4 Discussion

### 4.1 Patient characteristics

#### 4.1.1 Age at fistula repair

The age at fistula repair was frequently studied but generally showed no clear association with outcome. Gray et al. demonstrated that younger age was associated with better outcomes (mean 49 years vs 68 years; p < 0.05) (11).

#### 4.1.2 Body mass index

The evidence to support the role of body mass index (BMI) was limited. One study found that underweight women had negative surgical repair outcomes (Odds ratio [OR], 0.49; 95% Confidence Interval [CI], 0.23-0.99, p = 0.048) (12). The undernutrition delays tissue healing process and hinder fistula closure.

#### 4.1.3 Number of living children

Literature revealed that women with ≥ 1 living children were more likely to have successful surgical repair outcomes, possibly due to lower psychological burden of the fistula (OR, 3.19; 95% CI, 1.09-9.64; p < 0.036) (12).

#### 4.1.4 Etiology

Evidence that etiology predicts outcome is weak and inconsistent. Multivariate analysis in one study demonstrated a threefold higher recurrence risk with obstetrical fistula uncertainty (OR, 3.03; 95% CI, 0.57-8.84; p < 0.03), though CI crossed 1.0, indicating uncertainty. The reported causes were prolonged obstetric labor (41%), gynecological surgery (43%), pelvic trauma (4.1%) and pelvic irradiation (1.3%) (16). Failure after obstetrical VVF repair has been linked to perifistula fibrosis, circumferential fistula and prior fistula repair attempts (17, 20). On the contrary, one study did not found significant difference between obstetric and non-obstetric etiologies (p = 0.491) (8) and radiation-induced VVF did not achieved significance in some series (7, 26).

#### 4.1.5 Urinary tract infection

The sparse evidence showed that pre-operative urinary tract infection (UTI) mat increase recurrence risk. One multivariate analysis reported 2.72 fold increase recurrence risk with positive preoperative UTI (OR, 2.72; 95% CI, 0.69-12.1; p < 0.03) (16), but the wide CI makes this estimate imprecise.

### 4.2 Fistula characteristics

#### 4.2.1 Fistula size and complexity

Literature support a negative influence of large fistula size on the VVF repair outcomes. Fistula diameter > 1 cm was associated with recurrence (p < 0.001) (16, 21), and the recurrence risk was fivefold increased (OR, 5.01; 95% CI, 1.72-7.1) (16). Similarly, fistula size > 3 cm is associated with repair failure in multiple reports (7, 19). Kayondo et al. found that fistula size > 3 cm had six times higher odds of unsuccessful repair (OR, 6.0; 95% CI, 1.46-24.63; p < 0.01) (19). Likewise, other study reported similar findings for mean size (success with 1.4 cm vs failure with 2.8 cm; p < 0.05) (11). Other studies revealed poor outcomes with fistula size > 4cm (OR, 3.5; 95% CI, 1.4-8.9) (23), and fistula size > 2 cm (OR, 0.40; 95% CI, 0.18-0.85; p < 0.019) (12). Moreover, larger defects signifies greater tissue loss and frequently associated with fibrosis and urethral involvement, compounding surgical difficulty.

However, other studies had contradictory findings and found no significance between size and recurrence. Fistula size > 2cm (27), Loposso M (24): size > 6 cm (p < 0.423; OR, 1.18; 95% CI, 0.78-1.79) and Maaljars (28): >3 cm fistula size (p < 0.139; OR, 0.52; 95% CI, 0.22-1.24) found no significant association. Although the point estimate suggested increased risk but the confidence interval crossed unity, indicating statistical uncertainty.

#### 4.2.2 Fibrosis and tissue quality

Peri-fistula fibrosis, scarring, and poor tissue quality were repeatedly associated with negative prognostic factors. Multiple studies advocates the association of peri-fistula fibrosis and vaginal scarring with repair outcome, including multivariate analysis identifying the effect of vaginal scarring and fibrosis on the surgical results (OR, 2.67; 95%;CI, 1.58-4.50) (17), severe scarring with 12- fold higher failure risk (OR, 12.24; 95% CI, 1.52-98.30; p = 0.004) (19) and increased odds of unsuccessful fistula closure (OR, 4.4; 95% CI, 1.9-10.4) (23), (OR, 2.95; 95% CI, 1.31-6.62; p = 0.009) (24), (OR, 3.7; p = 0.0006) (29). Other studies also demonstrates the similar outcome (p= 0.005) (27), (OR, 4.2; 95% CI, 1.398-12.739; p = 0.01) (22) with wide CI, reflecting imprecision rather than a strong association, although the overall direction of effect remained consistent. A study reported no significant effect (OR, 0.52; 95% CI, 0.22-1.24; p = 0.139) (28). The subjective grading of fibrosis in multiple cohorts highlights the need for standardized severity assessment tools.

#### 4.2.3 Urethra and bladder neck involvement

The data supports an antagonist influence of urethra and bladder neck involvement on VVF repair outcomes. The involvement of continence mechanism was associated not only with recurrence but also as post-operative residual incontinence even after successful fistula repair in patients with urethra or bladder neck inclusion. Several studies demonstrated the affiliation between urethra/bladder neck involvement and unsuccessful fistula repair outcomes (p = 0.015) (27) and 60% lower odds of achieving a closed and continent outcome (OR, 0.41; 95% CI, 0.22-0.75; p =0.004) (12). Furthermore, study found an independent affiliation between urethral involvement, including circumferential damage and failure to close (OR, 1.56; 95% CI, 0.94-2.59) (17) with nine times increased odds of closure failure (OR, 9.333; 95% CI, 2.23-39.12; p =0.004) and residual stress incontinence in other series (OR, 10.50; 95% CI, 1.39-79.13; p < 0.05) (19). The result suggested an association between involvement of urethra and recurrence of VVF repair but wide CI leads to limited precision. The multivariate analysis revealed that distance of fistula edge from external urethral meatus (EUM) < 1.5 cm was associated with repair failure (OR, 0.08; 95% CI, 0.02-0.25; p <0.001) and residual incontinence (OR, 0.12; 95% CI, 0.05-0.30; p <0.001). Hence, anatomical closure only may not be the best predictor to define success, functional outcomes including post-operative continence should also be considered (28). Unlike, one study demonstrated non-significant effect of urethra/bladder neck destruction on the fistula closure outcomes (p>0.05) (29).

#### 4.2.4 Prior failed repair

The evidence of previous repair on the fistula closure is inconsistent. The new studies suggested that prior VVF repair were not associated with recurrence outcomes (7, 16), furthermore, no statistically significant difference was found on logistic regression (26), (OR, 0.83; 95% CI, 0.58-1.19; p = 0.307) (27), (OR, 1.66; p = 0.0529) (29). Another study discussed the subjective “difficulty of repair” metric and found non-significant reduced odds (OR, 0.57; 95% CI, 0.14-1.96; p = 0.38). Henceforth, the point estimate suggested increased risk but the CI crossed unity, indicating statistical uncertainty. The difficulty of repair was based on quality of tissue, degree of scarring, size, and location of the fistula (18). On contrast, primary unsuccessful VVF repair was significantly associated with subsequent closure failure (OR, 4.7; 95% CI, 2.2-10.0; p <0.001) and with incontinence (OR, 2.8; 95% CI, 1.3-5.9; p <0.001) (23).

#### 4.2.5 Number of fistulae

Two multivariate analysis demonstrated that multiple fistulas were considered as independent predictors of poor outcome (OR, 8.2; 95% CI, 2.1-32.5; p =0.003) (8) and of 4 times higher recurrence risk (OR, 4.05; 95% CI, 0.52-12.4; p=0.05) (16). The latter estimate is imprecise with a CI crossing unity probably reflecting small sample size.

#### 4.2.6 Fistula classification systems

The components of several classification systems have been correlated variably with surgical outcomes. Using the Goh classification, the type and size of fistula did not differ significantly in fistula closure rates (p > 0.7), but type 1 fistula females were significantly continent after repair and type 4 being least likely (p < 0.01) (20). Another study reported anatomical closure and functional continence rates of 90% and 100% respectively for type 1 fistula, but declining progressively to type 4 (11).

The Waaldijk classification did not significantly influence the surgical outcome with statistical uncertainty as CI crossed unity (OR, 1.06; 95% CI, 0.88-1.27; p =0.550) (24).

The Panzi Score showed that VVF females with score 3 were associated with highest odds of surgical failure with strong positive association (OR, 4.13; 95% CI, 1.73-9.85; p =0.001), with each 1 point increment associated with 65% rise in odds of surgical failure (25).

### 4.3 Peri-operative characteristics

#### 4.3.1 Surgical approach and interposition

Transvaginal repair was common technique used for obstetric fistulas (near bladder neck) with consistently high success in appropriately selected cases (16). Abdominal (open/laparoscopic) approaches were more frequently reported for supratrigonal or complex iatrogenic fistulas. The multiple studies suggested that while anatomical closure rates were comparable across approaches, though early functional bladder outcomes may differed (p=0.41) (17), (p>0.05) (30), (p=0.13) (7), (p=0.069) (16). Laparoscopic VVF repair had high cure rates of 98% cure rates with mean follow up 17.3 months (10).

The vascularized interposition flaps such as Martius or omental flaps appear beneficial in complex, recurrent, or irradiated cases, though randomized data are limited. The global data showed that the success rate significantly increased to 94% by interposing omentum (p=0.002) (7) and reduced odds of failure (OR, 0.3; 95% CI, 0.1-0.7; p =0.02) (16). However one study demonstrated 98% cure without omentum interposition. Furthermore, same study demonstrated the positive association of martius flap used during vaginal VVF repair but with unadjusted association (p=0.038) (31). However, other study did not showed significant benefit with Martius flap interposition (16).

#### 4.3.2 Fistula closure technique

The only study determining the single vs double layer fistula closure found no significant association with recurrence and the CI crossed unity, indicating statistical uncertainty (OR, 1.17; 95% CI, 0.73-1.87) (17). However, other series reported excellent outcome with 3- layer repair technique (98% cure) (10), but comparative evidence is limited.

## 5 Limitations

This review has several limitations. The literature search was restricted to PubMed/MEDLINE and manual reference screening, so relevant studies indexed exclusively in other databases may have been missed. Most included studies were retrospective cohorts or case series, limiting causal inference and increases susceptibility to selection bias, recall bias, and uncontrolled confounding. Moreover, several included studies evaluated mixed genitourinary fistula populations, including urethrovaginal fistulas (UVF). Concurrent UVF may complicate surgery as this necessitates reconstructive procedures. Although UVF was reported by some studies (7, 19, 21), its occurrence was inconsistently reported preventing determination of how UVF influences recurrence after VVF surgery, thereby limiting the specificity of findings for vesicovaginal fistulas.

There was substantial heterogeneity in follow-up of the selected studies. Some studies lacked clear follow up information and others used different follow-up interval for reporting outcomes. As recurrence can occur late, variable and short follow up may impact on the success and the recurrence rate. However, setting a uniform minimum follow-up duration would have made the results comparable; but this would have excluded many eligible studies. Future prospective studies should adopt uniform follow-up protocols to facilitate more accurate assessment of recurrence following VVF repair.

## 6 Conclusion

This systematic review has identified the commonly reported predictors for vesicovaginal fistula repair recurrence. In 7 of 9 studies, large fistula size (>3 cm) was technically difficult to close due to tension at repair site and higher recurrence rate. Similarly, seven out of eight studies demonstrated peri-fistula fibrosis and scarred tissues heal poorly and reduces the success rate. A data of six in 7 studies demonstrated that prior VVF surgeries did not raised the chances of recurrence. The influence of previous failed repair remains uncertain, with conflicting results reported across studies. The urethra/bladder neck involvement was associated with poor fistula closure rates and persistent incontinence (depicted in 5 studies). Complex fistulas on various classification systems correlated with worse outcomes (3 studies). Similarly, 2 studies described about the unsuccessful repair outcomes in multiple fistulas or absence of flap interposition (**Table 3**). A limited data showed that untreated pre-operative UTI as a potential risk factor. Despite variability in study design and population characteristics, primary repair success rates exceeded 80-95%. However, preoperative risk stratification based on standardized classification systems such as the Goh and Panzi and recurrence risk factors may optimize outcomes. Protective factors for recurrence included early intervention, management at specialized fistula centers, and use of interposition flaps in complex cases.

**Table 3:** Summary of predictors of VVF repair failure across studies.

Risk factor / predictor	Number of studies reporting	Representative studies	Clinical interpretation
Large fistula size	7	Ayed 2006, Lewis 2009, Nardos 2009, Zhou 2017, Bernard 2019, Zeleke 2025, Zaghbib S	Larger defects are technically difficult to close and have higher tension at repair site
Previous repair attempts	6	Bernard 2019, Maljaars 2023, Ockrim 2009, Colenbrander 2021, Ayed M 2006, Lewis 2009	Results were uncertain with conflicting results
Severe fibrosis / scarring	7	Ayed 2006, Kayondo 2011, Lewis 2009, Nardos 2009, Ockrim 2009, Sjøveian 2011, Arrowsmith 1994	Scarred tissue heals poorly and reduces surgical success
Urethral involvement	5	Nardos 2009, Kayondo 2011, Zeleke 2025, Maljaars 2023, Arrowsmith 1994	Associated with complex fistula and continence issues
Radiation-induced fistula	3	Ockrim 2009, Colenbrander 2021, Ayed M 2006	Radiation damages tissue vascularity
Long duration of fistula	3	Kayondo 2011, Lewis 2009, Zeleke 2025	Chronic inflammation and fibrosis increase surgical difficulty
Complex fistula classification (Goh / Panzi)	3	Goh 2008, Beardmore-Gray 2017, Mukwege 2018	Higher stage correlates with worse outcomes
Multiple fistulas / complex anatomy	2	Colenbrander 2021, Zaghbib 2021	Requires advanced reconstructive techniques

## 7 Future directions

Additional research is necessary to validate recurrence predictors and risk factors for vesicovaginal fistula repair through prospective studies and predictive modeling. Improved imaging techniques for tissue evaluation and further research on flap techniques for vesicovaginal fistula repair can reduce recurrence rates, especially for high-risk iatrogenic and recurrent vesicovaginal fistulas. Studies should adhere to uniform outcome definitions and minimum follow up durations to identify late recurrences. Regional disparities in vesicovaginal fistula causes, including iatrogenic and obstetric causes in high- and low- and middle-income countries (LMICs), respectively, can be addressed through global guidelines to optimize vesicovaginal fistula treatment worldwide.
